# Rehabilitation following rotator cuff repair: A survey exploring clinical equipoise among surgical members of the British Elbow and Shoulder Society

**DOI:** 10.1177/17585732211059804

**Published:** 2021-12-01

**Authors:** Bruno Mazuquin, Marcus Bateman, Alba Realpe, Steve Drew, Jonathan Rees, Chris Littlewood

**Affiliations:** 1 Department of Health professions, Faculty of Health and Education, 5289 Manchester Metropolitan University, Manchester, UK; 2 Derby Shoulder Unit, University Hospitals Derby & Burton NHS Foundation Trust, Derby, UK; 3 Population Health Sciences, 1980 University of Bristol, Bristol, UK; 4 University Hospitals Coventry and Warwickshire, Coventry, UK; 5 Nuffield Department of Orthopaedics, Rheumatology and Musculoskeletal Science, University of Oxford and NIHR Oxford Biomedical Research Centre, Oxford, UK

**Keywords:** rotator cuff repair, rehabilitation, survey, equipoise

## Abstract

**Background:**

We investigated clinical equipoise across surgical members of the British Elbow and Shoulder Society (BESS) in relation to rehabilitation following rotator cuff repair.

**Method:**

An online survey explored clinical equipoise regarding early patient-directed versus standard rehabilitation after rotator cuff repair to inform the design of a national randomised controlled trial (RCT). It described different clinical scenarios relating to patient age, tear size, location and whether other patient-related and intra-operative factors would influence equipoise.

**Results:**

76 surgeons completed the survey. 81% agreed/ strongly agreed that early mobilisation might benefit recovery; 57% were neutral/ disagreed that this approach risks re-tear. 87% agreed/ strongly agreed that there is clinical uncertainty about the effectiveness of different approaches to rehabilitation. As age of the patient and tear size increased, the proportion of respondents who would agree to recruit and accept the outcome of randomisation reduced, and this was compounded if subscapularis was torn. Other factors that influenced equipoise were diabetes and non-secure repair.

**Conclusion:**

Surgical members of BESS recognise uncertainty about the effectiveness of different approaches to rehabilitation following rotator cuff repair. We identified a range of factors that influence clinical equipoise that will be considered in the design of a new RCT.

## Introduction

Rotator cuff repair surgery is a common intervention indicated for patients with symptomatic rotator cuff tears.^1^ After surgery, patients undergo rehabilitation and despite its major role in a patient's recovery, there is clinical uncertainty regarding the optimal approach to rehabilitation after rotator cuff repair.^2^ Cautious approaches, where patients use a sling for over a month and only passive movements are allowed in the initial postoperative stages are common in current clinical practice.^3^ A recent survey with physiotherapists reported that the most frequent length of immobilisation after rotator cuff repair is four to six weeks.^3^ The rationale for more conservative approaches is the perceived risk of tendon re-tear if early mobilisation is introduced.^2^

We have recently completed a National Institute for Health Research funded pilot and feasibility randomised controlled trial (RCT) that recruited 73 participants across five NHS hospitals comparing early patient-directed rehabilitation (discarding the shoulder sling as soon as possible following surgery and moving as pain allows) with standard rehabilitation (sling use for four weeks following surgery).^4^ The pilot and feasibility RCT met the predefined success criteria and suggested that a fully powered RCT is feasible with minor amendments to the research design. One of the main issues observed in the pilot RCT was the withdrawal of 5/37(13.5%) patients randomised to early patient-directed rehabilitation due to surgeons perceived risk of re-tear. Treatment preference and lack of clinical equipoise may hinder patient recruitment to an RCT, adherence to the allocated treatment and even early closure of a trial.^5,6^ The concept of equipoise has been defined as, based on current knowledge, the patient being neither advantaged nor disadvantaged if they were to receive any of the treatments being tested. Equipoise can be sub-divided into individual equipoise, where there is uncertainty at the level of the individual clinician, and community or clinical equipoise, where there is uncertainty in the clinical community about which treatment is best. It is typically the latter, community equipoise, upon which recruitment to RCTs is grounded.^7^

To help with refining the design of a future fully powered RCT, in this current study our objective was to explore clinical equipoise among surgical members of the British Elbow and Shoulder Society (BESS) in relation to early mobilisation of the shoulder following rotator cuff repair, in the context of different clinical scenarios and patient-related and surgical factors.

## Method

An online survey was hosted by Online Surveys (https://www.onlinesurveys.ac.uk). The survey was designed with reference to different clinical scenarios relating to the age of the patient, size and location of the rotator cuff tear and whether other factors, e.g. smoking status, diabetes, non-secure surgical repair, would influence a surgeon's decision to recruit or withdraw participants following randomisation (Supplementary material 1). For the clinical questions, surgeons were asked to specify their agreement on a 5-point Likert scale (strongly agree, agree, neutral, disagree or strongly disagree) or as Yes/No/Unsure. The survey was developed and piloted across the study team. Based on the pilot testing, the survey took less than 10 min to complete. It was distributed by email to BESS members with a reminder sent after two weeks. The survey was open for four weeks (11th March 2021 to 8th April 2021). A favourable ethical opinion was gained from the Manchester Metropolitan University Research Ethics Committee (Ref: 28603).

### Participants

Surgeon members of the British Elbow and Shoulder Society.

### Data analysis

Data was exported from Online Surveys to Microsoft Excel and analysed descriptively.

## Results

Seventy-six responses were received. Regarding surgeons’ clinical experience, 43.4% (33/76) reported less than 10 years, 13.2% reported between (10/76) 10 to 15 years and 43.4% (33/76) reported more than 15 years of clinical experience. The reported number of rotator cuff repairs performed monthly was mean = 3.7 (SD = 3), median = 3 and mode = 3.

The following sections report the responses to the statements and questions posed in the survey.

**It is possible that early patient-directed rehabilitation might benefit recovery after rotator cuff repair.**

**Figure 1.**
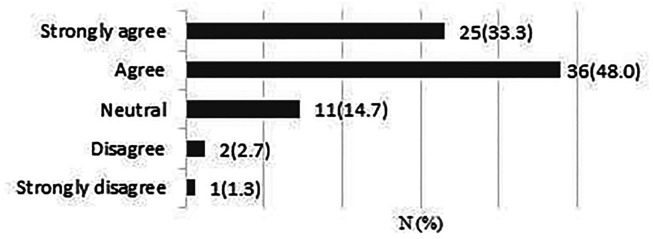
It is possible that early patient-directed rehabilitation might benefit recovery after rotator cuff repair.

A total of 75 responses were obtained. 61 respondents (81.3%) strongly agreed/agreed that early rehabilitation might be beneficial for patients recovery after rotator cuff repair (Figure 1).

**Early patient-directed rehabilitation risks re-tear following rotator cuff repair.**

**Figure 2.**
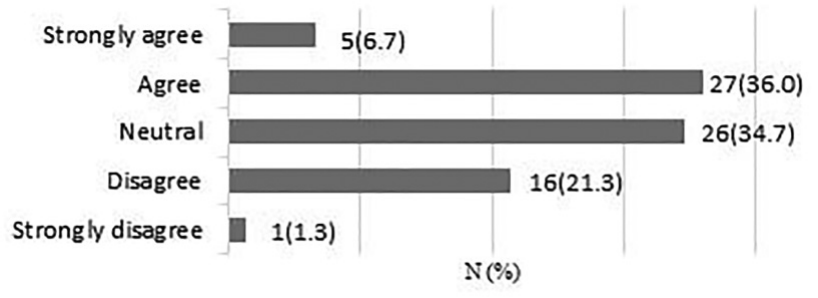
Early patient-directed rehabilitation risks re-tear following rotator cuff repair.

A total of 75 responses were obtained. 42 respondents (56%) were neutral or disagreed that early rehabilitation risks re-tear (Figure 2).

**There is clinical uncertainty about the effectiveness of different approaches to rehabilitation following rotator cuff repair surgery.**

**Figure 3.**
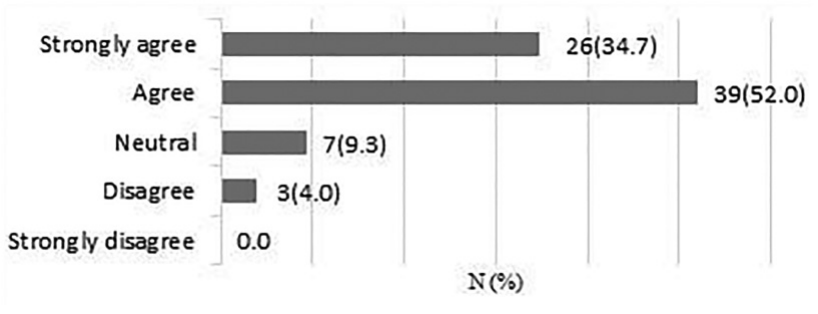
There is clinical uncertainty about the effectiveness of different approaches to rehabilitation following rotator cuff repair surgery.

A total of 75 responses were obtained. 65 respondents (86.7%) strongly agreed/agreed that there is clinical uncertainty regarding the effectiveness of different approaches to rehabilitation after surgery (Figure 3).

**A large randomised controlled trial (*n* = 600) comparing early patient-directed versus standard rehabilitation following rotator cuff repair is feasible within the UK NHS.**

**Figure 4.**
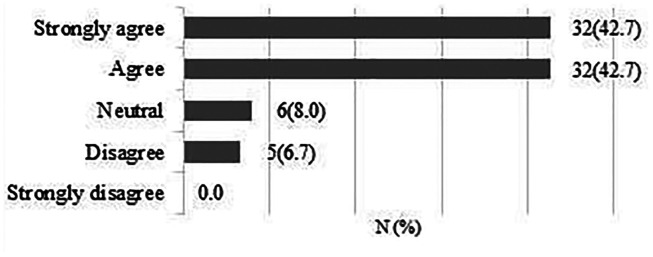
A large randomised controlled trial (*n* = 600) comparing early patient-directed versus standard rehabilitation following rotator cuff repair is feasible within the UK NHS.

A total of 75 responses were obtained. 64 respondents (85.4%) strongly agreed/agreed that a large RCT is feasible within the UK NHS (Figure 4).

**I would be interested in taking part in a fully powered randomised controlled trial.**

**Figure 5.**
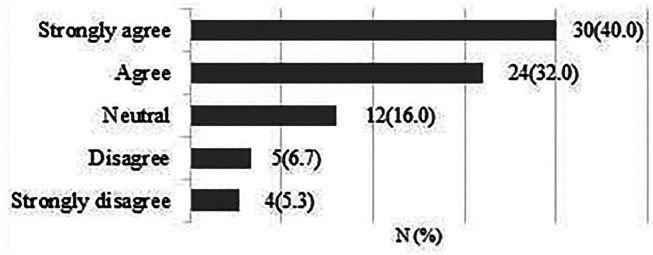
I would be interested in taking part in a fully powered randomised controlled trial.

A total of 75 responses were obtained. 54 respondents (72%) strongly agreed/agreed that they would be interested in taking part in the RCT (Figure 5).

### Clinical scenarios

 Table 1 describes the extent to which surgeons would agree to recruit and accept the outcome of the randomisation in different clinical scenarios. For a 55-years old with a small tear and intact subscapularis, 89.6% strongly agreed/agreed that they would recruit and accept the outcome of the randomisation. This percentage decreased to 47.3% if a 55-years old patient presented a large tear and intact subscapularis. If the subscapularis was torn, the percentages of those who strongly agreed/agreed decreased to 67.1% and 29%, respectively, for a 55-years old with a small and large superior/posterior tears.

**Table 1.** Surgeons’ extent of agreement to recruit and accept the outcome of the randomisation in different clinical scenarios.

	Subscapularis involvement													
Patient's characteristics	Intact							Torn						
Posterior/superior cuff involvement	Strongly agree	Agree	Neutral	Disagree	Strongly disagree	Missing	Total	Strongly agree	Agree	Neutral	Disagree	Strongly disagree	Missing	Total
	N (%)	N (%)	N (%)	N(%)	N (%)	N (%)	N (%)	N (%)	N (%)	N (%)	N(%)	N (%)	N (%)	N (%)
Small tear														
55 years old	44(58.0)	24(31.6)	3(3.9)	1(1.3)	2(2.6)	2(2.6)	76(100)	24(31.6)	27(35.5)	10(13.2)	11(14.5)	2(2.6)	2(2.6)	76(100)
65 years old	42(55.3)	25(32.9)	5(6.6)	0(0)	2(2.6)	2(2.6)	76(100)	23(30.3)	26(34.2)	13(17.1)	10(13.2)	2(2.6)	2(2.6)	76(100)
70 years old	38(50.0)	25(32.9)	4(5.3)	3(3.9)	3(3.9)	3(3.9)	76(100)	19(25.0)	25(32.9)	14(18.4)	13(17.1)	2(2.6)	3(3.9)	76(100)
Medium tear														
55 years old	27(35.5)	34(44.7)	5(6.6)	6 (7.9)	2(2.6)	2(2.6)	76(100)	19(25.0)	22(28.9)	13(17.1)	16(21.1)	3(3.9)	3(3.9)	76(100)
65 years old	25(33.0)	35(46.1)	8(10.5)	3(3.9)	3(3.9)	2(2.6)	76(100)	16(21.1)	23(30.3)	14(18.4)	17(22.3)	4(5.3)	2(2.6)	76(100)
70 years old	23(30.3)	32(42.1)	10(13.2)	6(7.9)	3(3.9)	2(2.6)	76(100)	14(18.4)	22(28.9)	16(21.1)	18(23.7)	4(5.3)	2(2.6)	76(100)
														
Large tear														
55 years old	9(11.8)	27(35.5)	14(18.4)	19(25.0)	5(6.6)	2(2.6)	76(100)	6(7.9)	16(21.1)	13(17.1)	30(39.5)	9(11.8)	2(2.6)	76(100)
65 years old	8(10.5)	22(28.9)	21(27.6)	18(23.7)	5(6.6)	2(2.6)	76(100)	6(7.9)	13(17.1)	14(18.4)	31(40.8)	10(13.2)	2(2.6)	76(100)
70 years old	8(10.5)	19(25.0)	15(19.7)	25(33.0)	7(9.2)	2(2.6)	76(100)	6(7.9)	11(14.5)	15(19.7)	31(40.8)	11(14.5)	2(2.6)	76(100)
Intact														
55 years old								13(17.1)	30(39.5)	16(21.1)	11(14.5)	3(3.9)	3(3.9)	76(100)
65 years old								12(15.8)	31(40.7)	17(22.4)	10(13.2)	4(5.3)	2(2.6)	76(100)
70 years old								12(15.8)	23(30.3)	20(26.3)	14(18.4)	5(6.6)	2(2.6)	76(100)

For a small tear and intact subscapularis, the percentage of surgeons who strongly agreed/agreed with recruiting and accepting the outcome of randomisation in the trial decreased from 89.6% for a 55-years old to 82.9% for a 70-years old. For a large tear and intact subscapularis, the percentage of surgeons who strongly agreed/agreed decreased from 47.3% for a 55-years old to 35.5% for a 70-years old.

### Patient-related factors and intra-operative findings

#### Patient is a regular smoker

**Figure 6.**
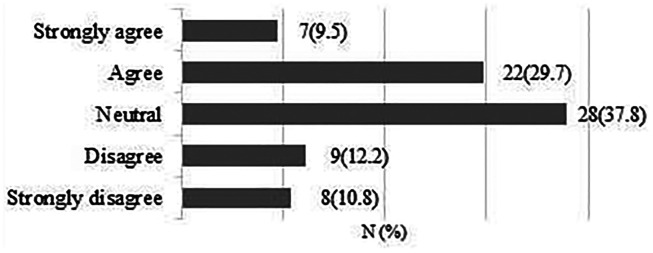
Patient is a regular smoker.

A total of 74 responses were obtained. 29 respondents (39.2%) strongly agreed/agreed that if the patient been a regular smoker it would influence their decision to recruit and accept the outcome of the randomisation (Figure 6).

#### Patient reports alcohol intake over recommended limits

**Figure 7.**
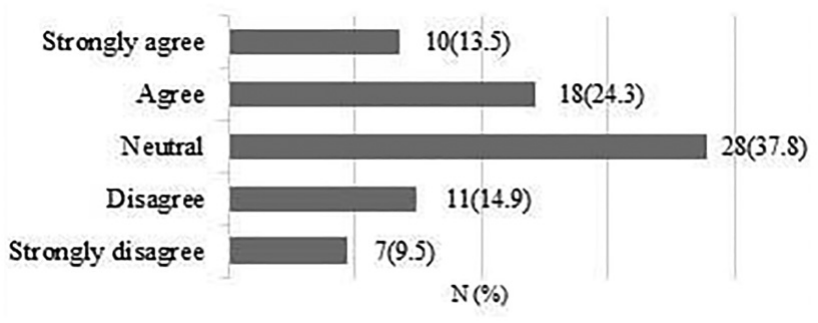
Patient reports alcohol intake over recommended limits.

A total of 74 responses were obtained. 28 respondents (37.8%) strongly agreed/agreed that the patient reporting alcohol intake over recommended limits would influence their decision to recruit and accept the outcome of the randomisation (Figure 7).

#### Diabetes

**Figure 8.**
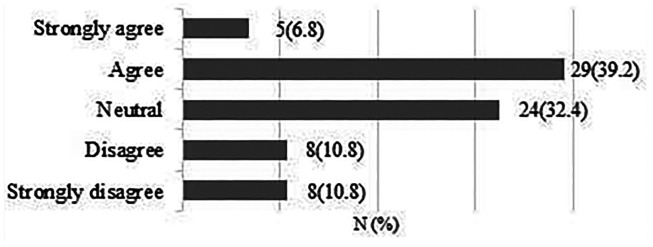
Patient has diabetes.

A total of 74 responses were obtained. 34 respondents (46%) strongly agreed/agreed that the patient having diabetes would influence their decision to recruit and accept the outcome of the randomisation (Figure 8).

#### Expectation of high functional demand post-surgery (sport or work)

**Figure 9.**
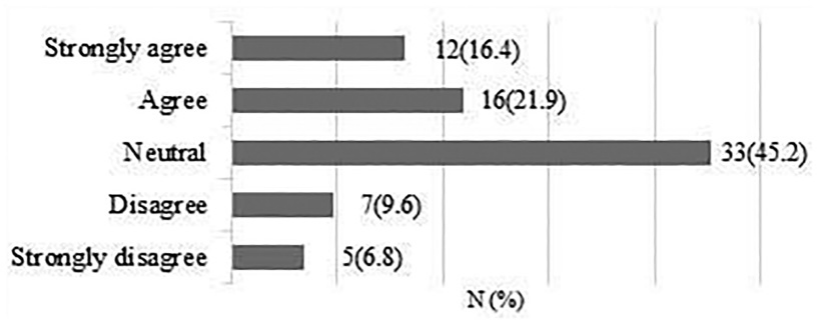
Expectation of high functional demand post-surgery (sport or work).

A total of 73 responses were obtained. 28 respondents (38.3%) strongly agreed/agreed that expectation of high functional demand post-surgery would influence their decision to recruit and accept the outcome of the randomisation 33 respondents (45.2%) were neutral (Figure 9).

#### Influence of intra-operative findings on the use of postoperative rehabilitation

The three main intra-operative findings that would influence surgeons’ decision to withdraw patients from the RCT were, in order, a repair that would not be regarded as secure, poor tissue quality and poor bone quality (Table 2).

**Table 2.** Influence of intra-operative findings on the decision to withdraw the patient from the RCT.

Intra-operative finding	Yes N (%)	No N (%)	Unsure N (%)	Missing N (%)	Total N (%)
Unsecure repair	52(68.4)	14(18.4)	7(9.2)	3(3.9)	76(100)
Tendon retraction	29(38.2)	34(44.7)	10(13.2)	3(3.9)	76(100)
Poor tissue quality	47(61.8)	19(25.0)	7(9.2)	3(3.9)	76(100)
Poor bone quality	39(51.3)	22(28.9)	11(14.5)	4(5.3)	76(100)
Biceps tenodesis required	7(9.2)	62(81.6)	4(5.3)	3(3.9)	76(100)

## Discussion

Reliability is an important property of a survey instrument. We did not evaluate the reliability of this current instrument and so do not know if surgeons would respond differently if repeating this survey. We also received a limited number of responses from surgical members of BESS (76/583 = 13%) which means the findings might not be generalisable to the wider community of surgeons performing rotator cuff repair'.

We completed an online survey to investigate clinical equipoise among surgical members of BESS with regard to rehabilitation following rotator cuff repair. We found that there is clinical equipoise across the surgical community reflected in the range of different opinions, and that there is willingness to participate in a future national trial.

The majority of respondents strongly agreed or agreed that early patient-directed rehabilitation might be beneficial to a patient's recovery. In terms of trial design, this suggests that a superiority trial is plausible from a clinical perspective. When asked whether early patient-directed rehabilitation risks re-tear, we found a spread in opinion suggesting that there is community equipoise regarding the most effective approach to postoperative rehabilitation.^7^

Regarding tear characteristics, we found that the size of the tear and subscapularis involvement influenced respondents’ decisions as to whether they would recruit and accept the outcome of the randomisation in the RCT. Almost 90% of surgeons strongly agreed/agreed that they would recruit and accept the outcome of the randomisation for a 55-year old patient with a small tear and intact subscapularis; however, this percentage decreased by 42.3% if a large tear was present. Our survey findings in relation to tear size reflect the current body of evidence. A recent systematic review suggested that early rehabilitation may be beneficial to patients with no additional risk imposed to the repair integrity.^8^ However, only a minority of the RCTs that were included in the systematic review recruited patients with large tears. The RCT from Sheps, Silveira,^9^ the largest completed to date (*n* = 206), included patients with large tears in their sample. They compared early rehabilitation (sling for comfort and pain-free shoulder movement for six weeks) with standard rehabilitation (sling for six and only passive and active-assisted shoulder exercises for six weeks). Their results showed that early rehabilitation was not superior to standard rehabilitation for range of movement, pain, strength and quality of life on the long-term. They also found that the risk of re-tear was higher for patients with large tears, but this was not influenced by the type of postoperative rehabilitation protocol; both early and standard rehabilitation groups had a similar number of re-tear events at 12 months. However, definitive recommendations for patients with a large tear cannot be made based only on the findings from Sheps, Silveira.^9^ An RCT with a representative sample of all tear sizes is needed to determine the safety, clinical and cost-effectiveness of early rehabilitation following rotator cuff repair. The tear size should also be considered a stratification factor for randomisation.

Patients’ age seems to have less influence on surgeons equipoise than tear characteristics. The percentage of surgeons who strongly agreed/agreed with recruiting and accepting the outcome of randomisation decreased only by 6.7% from a 55-years old to a 70-years old patient with a small tear and intact subscapularis but decreased by almost 12% from a 55-years old to a 70-years old patient with a large tear and intact subscapularis. In terms of research design, this is an important factor to consider. Another RCT on shoulder surgery reported that surgeons who were not in equipoise did not recruit patients who were eligible.^5^ Patients who were eligible but were excluded tended to be older and with less severe problems.^5^ Our survey also suggests that other factors affecting rotator cuff repair healing rates commonly reported in the literature,^10^ i.e. diabetes, unsecure repair and tissue quality, would be the most common reasons for surgeons not recruiting and accepting the outcome of randomisation. The survey findings also suggest that there is a readiness for a definitive trial. 86.7% of surgeons strongly agreed/agreed that a large RCT is feasible within the UK NHS and 72% strongly agreed/agreed that they would be interested in taking part in a fully powered RCT. This is encouraging given that the expected sample size needed for the fully powered RCT is over 600 patients.

## Conclusion

There is uncertainty about the optimal approach to rehabilitation following rotator cuff repair. This was recognised by surgical members of BESS, who are also willing to participate in a future RCT to answer the research question of whether an early patient-directed rehabilitation is clinically effective compared to standard rehabilitation. We have identified a range of factors that influence clinical equipoise, and a range of different opinions across the community. This information will be factored into the design and in the training of clinicians taking part in a fully powered RCT to be undertaken in the UK NHS.

## Supplemental Material

sj-docx-1-sel-10.1177_17585732211059804 - Supplemental material for Rehabilitation following rotator cuff repair: A survey exploring clinical equipoise among surgical members of the British Elbow and Shoulder Society Click here for additional data file. Supplemental material, sj-docx-1-sel-10.1177_17585732211059804 for Rehabilitation following rotator cuff repair: A survey exploring clinical equipoise among surgical members of the British Elbow and Shoulder Society by Bruno Mazuquin, Marcus Bateman, Alba Realpe, Steve Drew, Jonathan Rees and Chris Littlewood in Shoulder & Elbow

## References

[1] Carr AJ Cooper CD Campbell MK , et al. Clinical effectiveness and cost-effectiveness of open and arthroscopic rotator cuff repair the UK Rotator Cuff Surgery (UKUFF) randomised trial. Health Technol Assess 2015; 19: 1–218. 10.3310/hta19800 PMC4781041 26463717

[2] Kane LT Lazarus MD Namdari S , et al. Comparing expert opinion within the care team regarding postoperative rehabilitation protocol following rotator cuff repair. J Shoulder Elbow Surg 2020; 29: e330–e337. 32386779 10.1016/j.jse.2020.01.097

[3] Littlewood C Mazuquin B Moffatt M , et al. Rehabilitation following rotator cuff repair: a survey of current practice (2020). Musculoskelet Care 2021; 19: 165–171. 10.1002/msc.1514 32939967

[4] Littlewood C Bateman M Butler-Walley S , et al. Rehabilitation following rotator cuff repair: a multi-centre pilot & feasibility randomised controlled trial (RaCeR). Clin Rehabil 2021; 35: 829–839. 33305619 10.1177/0269215520978859 PMC8191146

[5] Keding A Handoll H Brealey S , et al. The impact of surgeon and patient treatment preferences in an orthopaedic trauma surgery trial. Trials 2019; 20: 570. 31533863 10.1186/s13063-019-3631-x PMC6751812

[6] Minns Lowe CJ Moser J Barker KL . Why participants in The United Kingdom rotator cuff tear (UKUFF) trial did not remain in their allocated treatment arm: a qualitative study. Physiotherapy 2018; 104: 224–231. 29361297 10.1016/j.physio.2017.09.002

[7] Rooshenas L Elliott D Wade J , et al. Conveying equipoise during recruitment for clinical trials: qualitative synthesis of clinicians’ practices across six randomised controlled trials. PLoS Med 2016; 13: e1002147. 27755555 10.1371/journal.pmed.1002147 PMC5068710

[8] Mazuquin B Moffatt M Gill P , et al. Effectiveness of early versus delayed rehabilitation following rotator cuff repair: systematic review and meta-analyses. PLoS One 2021; 16: e0252137. 34048450 10.1371/journal.pone.0252137 PMC8162656

[9] Sheps DM Silveira A Beaupre L , et al. Early active motion versus sling immobilization after arthroscopic rotator cuff repair: a randomized controlled trial. Arthroscopy 2019; 35: 749–760. e2. 30827428 10.1016/j.arthro.2018.10.139

[10] Zhao J Luo M Pan J , et al. Risk factors affecting rotator cuff retear after arthroscopic repair: a meta-analysis and systematic review. J Shoulder Elbow Surg 2021; 30: 2660–2670. 34089878 10.1016/j.jse.2021.05.010

