# Supplementary material for: Rehabilitation following rotator cuff repair: A survey exploring clinical equipoise among surgical members of the British Elbow and Shoulder Society
Source: Shoulder Elbow. 2021 Dec 1;14(5):568–73. doi: 10.1177/17585732211059804 (PMC9527485; doi:10.1177/17585732211059804)
Supplement: sj-docx-1-sel-10.1177_17585732211059804 - Supplemental material for Rehabilitation following rotator cuff repair: A survey exploring clinical equipoise among surgical members of the British Elbow and Shoulder Society [file sj-docx-1-sel-10.1177_17585732211059804.docx]

**Supplementary material 1.** Online questionnaire

SECTION ONE

1. How many years of experience do you have working as a shoulder surgeon?

- < 5 years

- 5 to < 10 years

- 10 to > 15 years

- 15 to < 20 years

- 20 years or more

2. On average, how many rotator cuff repairs do you perform per month?

___________

3. Please indicate the extent to which you agree or disagree with the following statements:

|  |  | Strongly agree | Agree | Neutral | Disagree | Strongly disagree |
| --- | --- | --- | --- | --- | --- | --- |
| 1 | It is possible that early patient-directed rehabilitation might benefit recovery after rotator cuff repair. |  |  |  |  |  |
| 2 | Early patient-directed rehabilitation risks re-tear following rotator cuff repair. |  |  |  |  |  |
| 3 | There is clinical uncertainty about the effectiveness of different approaches to rehabilitation following rotator cuff repair surgery. |  |  |  |  |  |
| 4 | A large randomised controlled trial (n=600) comparing early patient-directed versus standard rehabilitation following rotator cuff repair is feasible within the UK NHS. |  |  |  |  |  |
| 5 | I would be interested in taking part in the RaCeR II randomised controlled trial. |  |  |  |  |  |

SECTION TWO

**In RaCeR II, participants will be recruited before surgery but then randomised after surgery to ensure that the repair takes place. The surgeon will always choose the most appropriate repair method depending on their practice. The comparison is early patient-directed rehabilitation versus standard rehabilitation. Please indicate the extent to which you would agree to recruit and accept the outcome of the randomisation in the following clinical scenarios:**

|  |  | Strongly agree | Agree | Neutral | Disagree | Strongly disagree |
| --- | --- | --- | --- | --- | --- | --- |
| 1 | 55 years old. Small (<1cm) posterior/superior tear with intact subscapularis. |  |  |  |  |  |
| 2 | 65 years old. Small (<1cm) posterior/superior tear with intact subscapularis. |  |  |  |  |  |
| 3 | 70 years old. Small (<1cm) posterior/superior tear with intact subscapularis. |  |  |  |  |  |
| 4 | 55 years old. Small (<1cm) posterior/superior tear with torn subscapularis. |  |  |  |  |  |
| 5 | 65 years old. Small (<1cm) posterior/superior tear with torn subscapularis. |  |  |  |  |  |
| 6 | 70 years old. Small (<1cm) posterior/superior tear with torn subscapularis. |  |  |  |  |  |
| 7 | 55 years old. Medium (1-3cm) posterior/superior tear with intact subscapularis. |  |  |  |  |  |
| 8 | 65 years old. Medium (1-3cm) posterior/superior tear with intact subscapularis. |  |  |  |  |  |
| 9 | 70 years old. Medium (1-3cm) posterior/superior tear with intact subscapularis. |  |  |  |  |  |
| 10 | 55 years old. Medium (1-3cm) posterior/superior tear with torn subscapularis. |  |  |  |  |  |
| 11 | 65 years old. Medium (1-3cm) posterior/superior tear with torn subscapularis. |  |  |  |  |  |
| 12 | 70 years old. Medium (1-3cm) posterior/superior tear with torn subscapularis. |  |  |  |  |  |
| 13 | 55 years old. Large (3-5cm including infraspinatus) posterior/superior tear with intact subscapularis. |  |  |  |  |  |
| 14 | 65 years old. Large (3-5cm including infraspinatus) posterior/superior tear with intact subscapularis. |  |  |  |  |  |
| 15 | 70 years old. Large (3-5cm including infraspinatus) posterior/superior tear with intact subscapularis. |  |  |  |  |  |
| 16 | 55 years old. Large (3-5cm including infraspinatus) posterior/superior tear with torn subscapularis. |  |  |  |  |  |
| 17 | 65 years old. Large (3-5cm including infraspinatus) posterior/superior tear with torn subscapularis. |  |  |  |  |  |
| 18 | 70 years old. Large (3-5cm including infraspinatus) posterior/superior tear with torn subscapularis. |  |  |  |  |  |
| 19 | 55 years old. Intact posterior/superior rotator cuff with torn subscapularis. |  |  |  |  |  |
| 20 | 65 years old. Intact posterior/superior rotator cuff with torn subscapularis. |  |  |  |  |  |
| 21 | 70 years old. Intact posterior/superior rotator cuff with torn subscapularis. |  |  |  |  |  |

If you wish to add further comments, then please do so in the box below:

SECTION THREE

**We want to know if other factors, pre-surgery, would influence your decision to recruit if you were taking part in RaCeR II. Please indicate the extent to which the following** **factors would influence your decision to recruit and accept the outcome of the randomisation:**

|  |  | Strongly agree | Agree | Neutral | Disagree | Strongly disagree |
| --- | --- | --- | --- | --- | --- | --- |
| 1 | Patient is a regular smoker |  |  |  |  |  |
| 2 | Patient reports alcohol intake over recommended limits |  |  |  |  |  |
| 3 | Patient is diabetic |  |  |  |  |  |
| 4 | Expectation of high functional demand post-surgery (sport or work) |  |  |  |  |  |

If any other patient-related factors would influence your decision to recruit and accept the outcome of the randomisation, please state below:

SECTION FOUR

**In RaCeR II, participants will be recruited before surgery but then randomised after surgery to ensure that the repair takes place. Please indicate whether you would consider the following factors, identified intra-operatively,** **as reasons to withdraw the patient from the study once surgery has been completed but prior to randomisation:**

|  |  | Yes | No | Unsure |
| --- | --- | --- | --- | --- |
| 1 | Surgical repair is not regarded as secure |  |  |  |
| 2 | Extent of tendon retraction |  |  |  |
| 3 | Poor tissue quality |  |  |  |
| 4 | Poor bone quality |  |  |  |
| 5 | Required biceps tenodesis |  |  |  |

If any other intra-operative findings would influence your decision to withdraw the patient, please state below:

If you would like to express an interest in participating in RaCeR II, subject to further information, then please insert your name and email address below:

Name: ________________________________________________________________

Email: _________________________________________________________________

If you are happy to be contacted in the future to discuss participation in an interview study to discuss the responses to this survey in more detail, then please insert your name and email address below:

Name: ________________________________________________________________

Email: _________________________________________________________________

THANK YOU FOR COMPLETING THIS SURVEY.
